# Glycosaminoglycans as Polyelectrolytes: Charge, Interactions, and Applications

**DOI:** 10.1002/cbic.202500418

**Published:** 2025-10-06

**Authors:** Gergo Peter Szekeres, Eunjin Moon, Johanna K. Elter, Bryce Roper, Jayachandran Narayanan Nair Kizhakkedathu, Matthias Ballauff, Rainer Haag, Kevin Pagel

**Affiliations:** ^1^ Institute of Chemistry and Biochemistry Freie Universität Berlin Altensteinstraße 23A 14195 Berlin Germany; ^2^ Department of Molecular Physics Fritz‐Haber‐Institut der Max‐Planck‐Gesellschaft Faradayweg 4‐6 14195 Berlin Germany; ^3^ Institute of Organic and Macromolecular Chemistry Friedrich‐Schiller‐Universität Jena Humboldtstraße 10 07743 Jena Germany; ^4^ Centre for Blood Research University of British Columbia 2350 Health Sciences Mall Vancouver V6T 1Z3 BC Canada; ^5^ School of Biomedical Engineering University of British Columbia 2350 Health Sciences Mall Vancouver V6T 1Z3 BC Canada; ^6^ Department of Pathology and Laboratory Medicine University of British Columbia 2350 Health Sciences Mall Vancouver V6T 1Z3 BC Canada

**Keywords:** charge–charge interactions, glycosaminoglycans, glycosaminoglycans mimetics, polyelectrolytes

## Abstract

Glycosaminoglycans (GAGs) are linear, negatively charged biopolymers that modulate complex biological processes, such as blood coagulation, immune regulation, or viral entry. Their sulfation pattern and chain length govern how strongly they bind to other physiologically relevant species. Most of these interactions rely on electrostatic forces facilitated by the strong polyanionic properties of GAGs; therefore, considering these from a polyelectrolyte vantage point can help understand how such charge‐based, often transient interactions contribute to physiological and pathological processes. While the different GAG classes share key electrostatic properties, they exhibit unique structural features that shape their function. Here, it is highlighted on how modern separation and analytical tools exploit the polyanionic character of GAGs to dissect subtle structural details. For these, the fundamental description of their charge–charge interactions is crucial. With this knowledge, modified GAGs, synthetic GAG mimetics, or GAG‐binding molecules can be designed that replicate or refine their key properties and show promise for therapeutic and biomedical applications. Altogether, recognizing the importance of GAGs as polyelectrolytes provides vital knowledge on how their charge distribution mediates crucial biomolecular interactions in health and disease, and thus it helps complete our knowledge on fundamentally important biopolymers.

## Graphical abstract

Glycosaminoglycans (GAGs) are linear, anionic biopolymers with their functions tightly linked to their charge. This review approaches GAGs from a polyelectrolyte perspective, shedding light on how the distribution of sulfate groups and, thus, negative charges, governs their interactions with other biomolecules. Understanding these charge‐driven principles enables their understanding, analysis, and the design of GAG‐based or GAG‐mimetic materials.

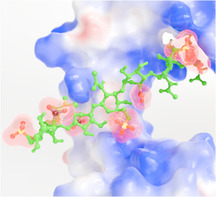

## 1 Introduction

Glycans are the most abundant biopolymers on Earth. Their remarkable structural diversity promotes a wide range of biological functions from energy storage to structural support. Among them, glycosaminoglycans (GAGs) form a distinct subgroup of linear glycans, classified by their repeating disaccharide building blocks and varying sulfation patterns. Each disaccharide typically consists of a hexuronic acid and an (*N*‐acetyl‐)amino sugar that carry negatively charged functional groups, that is, carboxyl and sulfate groups. These lend GAGs a pronounced polyanionic character that is central to understanding their biological activities in developmental processes, cellular signaling, and interactions with proteins and other biomolecules.

**Figure 1:**
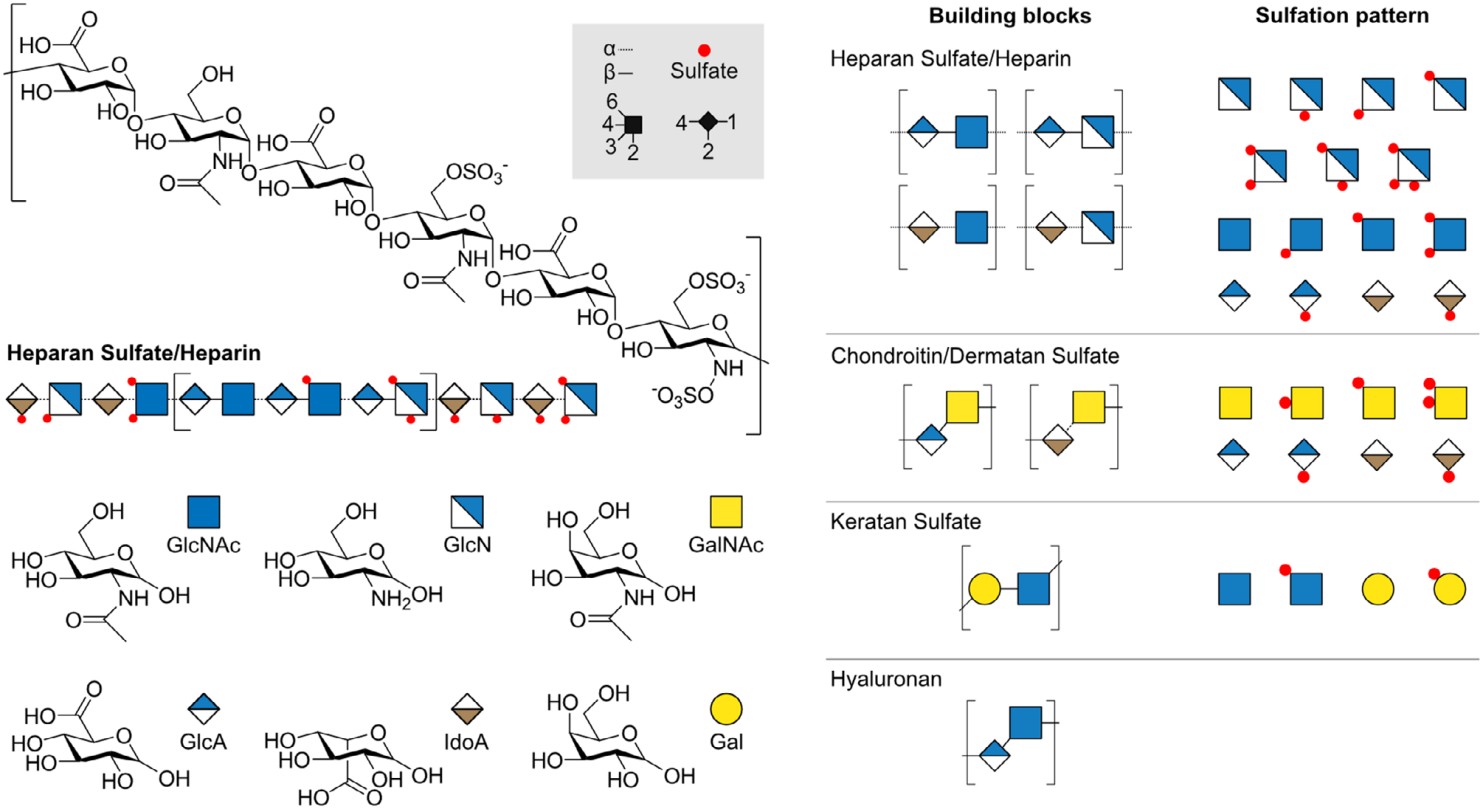
The structure and classification of GAGs. Glycan structures are generally visualized by the guidelines of a simplified system called the *Symbol Nomenclature for Glycans* (SNFG).^[^ ^200^ ^]^

GAGs are commonly divided into four classes based on their monosaccharide composition and connectivity (**Figure** **1**): heparan sulfate/heparin (HS/Hep), chondroitin‐ and dermatan sulfate (CS/DS), keratan sulfate (KS), and hyaluronic acid or hyaluronan (HA). As exceptions, KS lacks carboxyl groups, while HA is exclusively present in a non‐sulfated form. These features stand out when GAGs are considered from a polyelectrolyte vantage point (**Table** **1**). The pattern and degree of sulfation, in combination with the number of carboxyl groups, determine the charge density of a given GAG. These differences are essential for understanding GAG conformation, ionic interactions, and the balance of electrostatic forces that guide their interactions with counter‐ions and biological macromolecules. As shown in Table 1, the average degree of sulfation is also characteristic to the GAG class, with Hep being the highest‐sulfated species and HA being completely non‐sulfated.

**Table 1:** The average number of sulfate groups per disaccharide unit *n*_s_ and corresponding charge parameters *ξ* of different GAGs from various sources. In the cases where the sulfation levels are presented as percentages, the corresponding *n*_s_ was calculated. *ξ* was calculated based on Equations (1)‐(2) with a Bjerrum length of 0.71 nm at room temperature (i.e., the distance between two charges at which the electrostatic interaction is *kT*) and a charge–charge distance approximated from the disaccharide unit length of 1 nm divided by the average number of charged groups. Note that while KS is higher sulfated on average than HS, CS, and DS, it does not present carboxyl groups along the chains, which results in lower *ξ*.

GAG type	Source	n_s_/disaccharide	*ξ*/disaccharide
Heparin	Porcine mucosa,^[^ ^74^ ^,^ ^202^ ^]^ bovine intestinal mucosa,^[^ ^203^ ^]^ healthy human blood plasma^[^ ^203^ ^]^	1.78–2.47	1.97–2.46
Heparan sulfate	Bovine endothelium,^[^ ^204^ ^]^ rat liver‐derived cell line,^[^ ^204^ ^]^ rat hepatocyte,^[^ ^204^ ^]^ mouse Simian‐virus‐40‐transformed fibroblast cell line,^[^ ^204^ ^]^ mouse fibroblast tumor cell line,^[^ ^204^ ^]^ mouse bone marrow stroma,^[^ ^204^ ^]^ mouse fibroblast normal cells,^[^ ^204^ ^]^ mouse granulosa,^[^ ^204^ ^]^ Chinese hamster ovary cell line,^[^ ^204^ ^]^ human neuroblastoma,^[^ ^204^ ^]^ human skin fibroblast,^[^ ^204^ ^]^ human healthy liver,^[^ ^205^ ^]^ human non‐fibrotic peritumoral liver,^[^ ^205^ ^]^ human hepatocellular carcinoma^[^ ^205^ ^]^	0.57–1.25	1.11–1.60
Chondroitin sulfate	Bovine cartilage,^[^ ^206^ ^]^ porcine cartilage,^[^ ^206^ ^]^ porcine trachea,^[^ ^207^ ^]^ porcine intestinal mucosa,^[^ ^208^ ^]^ chicken cartilage,^[^ ^206^ ^]^ chicken keel cartilage,^[^ ^208^ ^]^ shark cartilage,^[^ ^206^ ^]^ skate cartilage,^[^ ^206^ ^]^ salmon,^[^ ^208^ ^]^ skipjack,^[^ ^208^ ^]^ monkfish,^[^ ^208^ ^]^ snake head^[^ ^208^ ^]^	0.80–1.20	1.28–1.56
Dermatan sulfate	Porcine skin,^[^ ^209^ ^]^ porcine intestinal mucosa,^[^ ^210^ ^]^ bovine mucosa,^[^ ^210^ ^]^ bovine trachea,^[^ ^208^ ^]^ monkfish^[^ ^208^ ^]^	0.91–1.11	1.36–1.50
Keratan sulfate	Human child costal cartilage,^[^ ^211^ ^]^ costal cartilage of human young adult with Marfan syndrome,^[^ ^211^ ^]^ human old adult costal cartilage,^[^ ^211^ ^]^ porcine nucleus pulposus,^[^ ^211^ ^]^ bovine cornea^[^ ^211^ ^,^ ^212^ ^]^	1.07–1.50	0.76–1.24
Hyaluronic acid	(Bacterial fermentation)	0	0.71

While at first glance, GAGs may look like a class of highly similar molecules, nuances in their chemical properties can lead to drastic changes in their structures and physiological behavior. Therefore, they can only be characterized in full, when simultaneously viewed from macromolecular structural, carbohydrate chemical, polyelectrolyte, and interactome vantage points. However, when one attempts to tackle this enormous undertaking, they must face the fact that each of these vantage points are further complicated. For example, their macromolecular structure, while linear, is highly flexible, thus intra‐ and intermolecular interactions play a key role in stabilizing a few, specific low‐energy conformers.^[^ ^1^, ^2^, ^3^ ^–^ ^4^ ^]^ Moreover, most GAGs, except for HA, are covalently bound to proteoglycans, which reduces their degree of freedom, concentrates them at the cell membrane, and integrates them in the large and highly complicated network of the glycocalyx. From the chemical point of view, it is easy to see how GAGs compose a very special class among all glycans: they are highly restricted in both the sugar unit composition as well as the connectivity between the monosaccharides; meanwhile, a highly varying pattern of sulfation propagates along the chains—a chemical modification that is not representative to other glycan classes. This sulfation pattern is the major reason behind their complex polyelectrolyte properties, which is often overlooked, potentially as a result of its superficial consideration, that is, a varying sulfation pattern will naturally lead (simply) to a varying polyelectrolyte character. Therefore, in this review, we approach GAGs from a polyelectrolyte vantage point to describe their structure–function relationship and shed light on how their strong anionic character plays a major role in their interactions with proteins, ions, and various other physiologically relevant species. This facilitates the explanation of how GAGs maintain their structure in the extracellular matrix, mediate cell signaling, and coordinate complex processes. These basic electrostatic principles, sometimes overlooked in more traditional carbohydrate‐focused views, reveal how GAGs bind in ways that affect both normal biological functions and diseases linked to GAGs.

One of the most prominent examples of GAG function is Hep's anticoagulant activity, which is extensively exploited in clinical practice.^[^ ^5^, ^6^, ^7^, ^8^ ^–^ ^9^ ^]^ While several anticoagulants are in use today, Hep remains popular, partly due to its high anticoagulant activity and because it can be neutralized instantly *via* simple protamine complexation.^[^ ^10^ ^,^ ^11^ ^]^ Beyond their medical relevance, GAGs contribute to a wide range of physiological phenomena. Abnormal GAG structures, in turn, have been linked to pathological states including cancer progression, metastasis, and neurodegenerative conditions such as Alzheimer's disease.^[^ ^12^, ^13^, ^14^, ^15^, ^16^, ^17^ ^–^ ^18^ ^]^ These associations stem from the fact that GAGs often facilitate cell signaling events,^[^ ^19^ ^,^ ^20^ ^]^ and modulate growth factors^[^ ^21^ ^,^ ^22^ ^]^ and chemokines,^[^ ^23^, ^24^, ^25^ ^–^ ^26^ ^]^ among others, through charge‐mediated interactions and higher–order complex formation.

An especially striking example of their biological importance is their role in viral infections.^[^ ^27^ ^,^ ^28^ ^]^ GAGs, particularly HS, are thought to be among the first host‐cell components encountering the virion. Electrostatic attraction between the negatively charged GAG chains and positively charged patches on viral proteins often initiates viral docking. This initial binding—hinting at GAGs as high‐avidity, low‐affinity receptors^[^ ^28^ ^,^ ^29^ ^]^—can induce conformational changes in viral proteins that expose membrane fusion domains required for viral entry.^[^ ^30^ ^,^ ^31^ ^]^ Exploiting this polyelectrolyte–based interaction for therapeutic purposes inspires the design of synthetic GAG analogs. These mimetics, which often have more defined sequences or higher charge density, can exhibit enhanced virus neutralization compared to their natural counterparts, as shown recently for SARS‐CoV‐2.^[^ ^32^ ^]^

GAG separation and their analysis both strongly rely on workflows adapted from proteomics. This was especially the case until 2008, that is, the Heparin Adulteration Crisis,^[^ ^33^ ^]^ which led to severe symptoms and death in clinical patients. The cause of this was the dilution of porcine Hep with semi‐synthetic over‐sulfated CS; while CS is also a GAG, structurally it differs from Hep, which evokes adverse immune reactions in patients who are treated with the contaminated drug. This led to the recognition that precise and routine analysis for Hep quality control must be developed, and transitioning to more specific and more sensitive analytical approaches for GAGs is of great importance.

The intrinsic polyelectrolyte character of GAGs also motivates diverse analytical and preparative techniques. From these, fundamental insights have emerged regarding counter‐ion release,^[^ ^34^ ^–^ ^37^ ^]^ ion‐pairing equilibria,^[^ ^38^ ^]^ and the overall charge regulation mechanisms in GAG‐based systems. Moreover, the possibility of GAG structural modification provides further opportunities to tune and investigate their polyelectrolyte properties. Modified GAGs and GAG mimetics have clear implications for therapeutic and biotechnological applications, including drug delivery,^[^ ^36^ ^,^ ^39^, ^41^, ^42^, ^43^ ^–^ ^43^ ^]^ biomaterial design,^[^ ^39^ ^,^ ^40^ ^,^ ^43^ ^,^ ^44^ ^]^ and antiviral strategies.^[^ ^32^ ^,^ ^45^ ^,^ ^46^ ^]^

## 2 GAG Structures

Each GAG class features unique disaccharide composition and sulfation that define its charge density and biological function (Figure 1). To better understand the functional diversity of GAGs, this section outlines their structural distinction and how it relates to their charge‐driven interactions.

### 2.1 Hep/HS

The disaccharide units of Hep and HS consist of 1,4‐linked *α*‐l‐iduronic acid (IdoA) or *β*‐d‐glucuronic acid (GlcA) and *β* *‐* d *‐*glucosamine (GlcN). These molecules exhibit extensive sulfation, with GlcA sulfated at the 2‐*O* position and GlcN either *N*‐acetylated (GlcNAc), or *N*‐sulfated (GlcNS). Additional sulfation can occur at the 6‐*O* and, less commonly, the 3‐*O* positions of GlcN.^[^ ^47^ ^]^ Despite their structural similarities, HS and Hep differ in chain length, IdoA content, and their sulfation pattern. HS chains contain 50–250 disaccharide units (20–100 kDa), whereas Hep is shorter (7–20 kDa).^[^ ^48^ ^]^ Hep is higher sulfated and contains > 70% IdoA, whereas HS has a lower sulfation and a higher proportion of GlcA (cf. Table 1). The sulfate groups in HS are not uniformly distributed but instead form three structural domains: *N*‐acetylated (NAc), alternating *N*‐acetylated/*N*‐sulfated (NAc/NS), and highly sulfated (NS) regions.^[^ ^49^ ^]^ These domains govern the selective binding of Hep to proteins.

### 2.2 CS/DS

CS consists of alternating *β*‐d‐GlcA (1,4‐linkage) and *N‐*acetyl‐galactosamine (GalNAc, 1,3linkage). CS chains typically contain 40–100 disaccharide units.^[^ ^50^ ^]^ The charge parameter of CS varies depending on its sulfation pattern, leading to different subtypes. CS‐A and CS‐C are monosulfated, primarily at the 4‐*O* (CS‐A) or 6‐*O* (CS‐C) positions of GalNAc, whereas CS‐B (also known as DS) contains 2‐*O* sulfated IdoA and 4‐*O* sulfated GalNAc. Other sulfated forms, for example, CS‐D, CS‐E, and CS‐K, further contribute to structure and charge heterogeneity.

### 2.3 KS

Unlike other GAGs, the building blocks of KS lack hexuronic acid: it consists of *β‐d‐*galactose (Gal) and *β*‐d‐GlcNAc linked *via* 1,3‐ and 1,4‐linkages. As it is exempt of carboxyl groups from the hexuronic acid monosaccharides, its negative charges solely originate from sulfate groups. KS sulfation varies depending on type and expression site, with sulfation primarily occurring at the 6‐*O* position of both Gal and GlcNAc.^[^ ^51^ ^]^

### 2.4 HA

HA is the only GAG without natural sulfation. Its disaccharide building block consists of *β*‐d‐GlcA and *β*‐d‐GlcNAc, connected by 1,3‐ and 1,4‐linkages. Despite lacking sulfation, HA is negatively charged by the carboxyl groups of GlcA that are deprotonated at physiological pH (pKa ≈ 3).^[^ ^52^ ^]^ Compared to other GAGs, HA forms significantly longer chains, with molar masses reaching into the MDa range.

### 2.5 Polyanionic Character

Cation binding and (partially) water retention are direct consequences of the polyanionic character of GAGs, which consequently dictates a variety of biochemical functions from intercellular signaling to the structural stabilization of the extracellular matrix. Variations of the polyanionic characters of GAGs, stemming from differences in sulfation pattern and the inherent presence of carboxyl groups, play a critical role in modulating macromolecular interactions and physiological outcomes. On a fundamental level, this defines their charge parameter (Table 1); meanwhile, from the physiological point of view, it influences molecular organization and their interactions. These interactions are essential for healthy biological functions, and to date, thousands of proteins have been identified that interact with GAGs.^[^ ^53^, ^54^, ^55^, ^56^ ^–^ ^57^ ^]^ It is, however, important to note that the observed interactions between GAGs and proteins may not all be functional, and the co‐localization of these molecules always needs to be taken into account.

The carboxyl group in hexuronic acids is deprotonated at physiological pH, which means that a GAG chain can carry a highly negative net charge even without sulfation (except KS). At the same time, sulfates have a pKa of ≈2.6,^[^ ^58^ ^]^ which leads to a constant deprotonated state under physiological conditions and increased polyanionic properties. Consequently, highly sulfated GAGs (e.g., Hep) exhibit the highest charge density, whereas HA, which lacks sulfation, has the lowest (cf. charge parameter ranges in Table 1). Since the localization of different GAG chains is specific in tissues, these charge variations must dictate their distinct physiological roles by shaping charge–charge interactions, binding specificity, and mechanical and structural properties. Highly sulfated Hep and HS engage in strong electrostatic interactions that modulate processes like coagulation, immune response, and growth factor signaling.^[^ ^59^ ^]^ In contrast, HA with a much weaker polyanionic character is linked to significantly fewer specific processes, and it is known to regulate tissue hydration, lubrication, and biomechanical properties of the extracellular matrix instead.^[^ ^60^ ^,^ ^61^ ^]^ However, higher sulfation does not always correlate with stronger protein binding affinity. Instead, specific sulfation patterns are critical in dictating selectivity: for example, CS‐E, one of the more sulfated CS, binds selectively to growth factors,^[^ ^62^ ^,^ ^63^ ^]^ and the principle of sequence specific electrostatics is increasingly recognized in the context of viral infections as well.^[^ ^28^ ^]^

## 3 Separation and Analysis

The separation and analysis of GAGs require advanced approaches. Contrary to proteins and nucleic acids, GAGs are biosynthesized in non‐template‐driven processes, which result in high chain length‐ and sulfation pattern heterogeneities as well as epimerization along the chains. These induce sample polydispersity, which often calls for complex analytical workflows.^[^ ^48^ ^,^ ^64^ ^–^ ^67^ ^]^ Moreover, GAGs are chemically highly similar molecules, and the differences in their measurable properties often lie below the resolution threshold of traditional analytical instrumentation. Therefore, GAGs have been historically neglected in bioanalytics, which makes it imperative to push the fields of separation and analysis forward by tailoring workflows specifically for GAGs.

In the separation and analysis of GAGs, it is essential to keep in mind that other than HA, GAGs are mostly found covalently bound to proteoglycans. On one hand, it helps their classification based on the fact that the class of GAGs bound to certain proteoglycans is often strictly regulated; meanwhile, on the other hand, it also means that for their sequencing and general quantitative analysis, GAGs must be cleaved first from the proteoglycans in a manner that preserves the full chain and its sulfation pattern. Then, as these molecules are anionic polyelectrolytes, it is straightforward to approach them with charge‐dependent techniques.

### 3.1 Separation

For a long time, different electrophoretic techniques have been used to separate different charges and lengths of GAGs, that is, their charge‐to‐mass ratio.^[^ ^68^, ^69^ ^–^ ^70^ ^]^ With the advances made in liquid chromatography (LC), GAG separation began to rely progressively more on this technique. Although LC separation is often preceded by depolymerization, hence omitting full‐chain information, it can give more in‐depth insight into the prevalent structural motifs and disaccharide building blocks within a sample than electrophoresis. Numerous chromatographic workflows have been developed for GAGs over the years relying on size exclusion,^[^ ^48^ ^,^ ^66^ ^,^ ^71^ ^]^ ion‐exchange,^[^ ^72^ ^,^ ^73^ ^]^ or even affinity columns when specific GAG sequences were in the focus (**Table** **2**).^[^ ^65^ ^,^ ^73^ ^]^ Recently, it was shown that high‐precision separation and analysis can be achieved by fluorescently labeling digested Hep disaccharides, which reports on sulfation degree and the ratio of disaccharides with different sulfation patterns.^[^ ^74^ ^]^

**Table 2:** General overview of different chromatography approaches for the separation of GAGs.

Method	Principle	Key advantages	Limitations	Compatibility
Ion‐exchange	Charge density	Separation by sulfation pattern, handles complex mixtures	Requires salt gradients, poor for unsulfated, and low‐sulfated KS	Hep/HS, CS/DS
Size exclusion	Molecular weight/size	Preserves native conformation, no harsh solvents	Low resolution, co‐elution of similar‐sized chains	HA, unfractionated Hep
Affinity	Specific binding	Isolates bioactive sequences	Expensive, limited to structures with known binders	In theory all, mostly used for Hep
Reverse‐phase ion pairing	Hydrophobicity and ion pairing	Compatible with MS, resolves sulfation isomers	Ion‐pairing agents suppress MS signal, risk of artifacts	All sulfated short sequences
Hydrophilic interaction	Polarity and charge	Compatible with MS, useful in resolution vs throughput compromise compared to ion‐exchange.	Long equilibration times, sensitive to water content	All

### 3.2 Analysis

**Figure 2:**
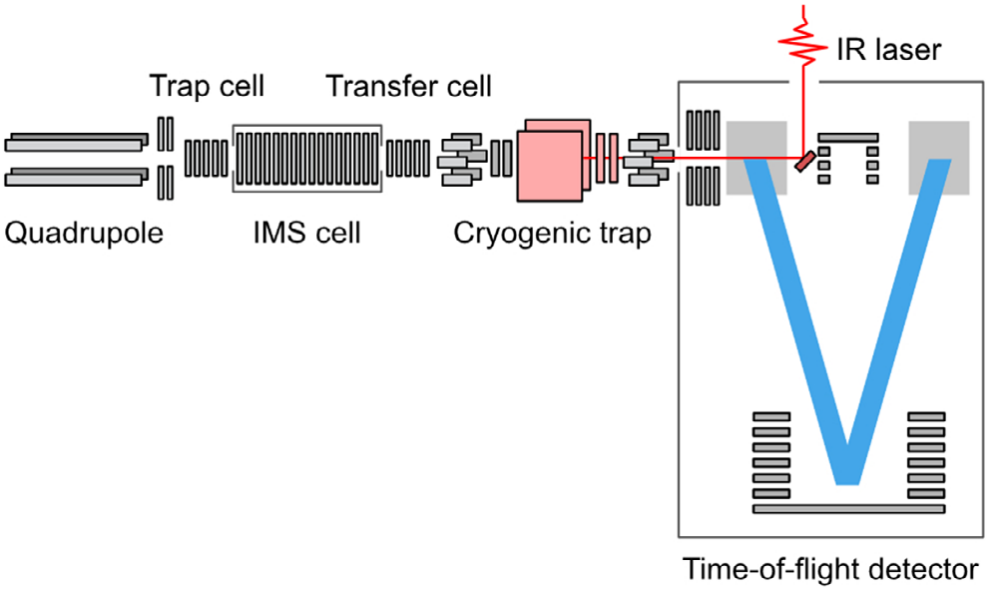
Schematic representation of an ion mobility‐mass spectrometer incorporating cryogenic gas‐phase IR spectroscopy. This instrument is a good demonstration of combining separation science and analysis in a single device: the quadrupole and ion mobility spectroscopy cell separate and select the analyte based on mass and shape, respectively, while the trap/transfer cells and the cryogenic ion trap with mid‐IR laser light respectively allow for determining the structural and spectroscopic features.

Sequence‐ and structure analytical techniques can also benefit from the charged properties of GAGs. Mass spectrometry (MS) is a prime example for that. From the homogeneous building blocks to heterogeneous size fractions, MS applications have been demonstrated for a wide variety of GAG structures.^[^ ^48^ ^]^ While many biomolecules are identified *via* fragmentation‐based tandem‐MS, sulfate loss occurs even at low activation, which limits the analysis. Combining MS with alternative techniques, such as ion mobility spectroscopy or gas‐phase infrared (IR) spectroscopy offers promising solutions to map the sulfation pattern. It was demonstrated that GAG disaccharides can be separately analyzed based on their sulfation pattern,^[^ ^74^ ^]^ and current progress in trapped ion mobility spectroscopy separation of glycans reaching the resolution of liquid chromatography^[^ ^75^ ^]^ suggests that the technique can be optimized further for identifying conformers of differently sulfated isobaric GAGs. Both ion mobility spectroscopy and MS allow for database construction: the former based on collision cross‐sections, while the latter based on the prevalent fragments in tandem‐MS applications. With their combination, it was possible to identify unknown structures by comparing their recorded data to those of known standards, and rare sulfation positions, for example, 3‐*O*‐sulfation could be identified.^[^ ^76^ ^]^ Recently, cryogenic gas‐phase IR spectroscopy approaches have been emerging, which point toward the possibility of the rapid and routine analysis of GAGs within a modified ion mobility‐MS setup (**Figure** **2**).^[^ ^77^ ^]^ Moreover, the results of cryogenic gas‐phase IR spectroscopy allow for fundamental insight into the GAG structures, which are essential in understanding GAG interactions, for example, at the cell membrane under physiological conditions.^[^ ^1^ ^,^ ^78^ ^]^

Among the more recent charge‐driven analytical techniques, nanopore sequencing of polyelectrolytes, especially nucleic acids, is gaining traction.^[^ ^79^ ^]^ Such instruments have more than an order of magnitude smaller footprint and are much simpler to operate than MS, which motivates the development of nanopore‐based polyelectrolyte sequencing workflows. In the past years, GAG sequencing based on nanopores was demonstrated at several instances.^[^ ^79^ ^,^ ^80^ ^]^

## 4 Charge–Charge Interactions

Charge–charge interactions play a vital role in the physiological function of biomolecules. Particularly, in the case of GAGs with a prevalent polyanionic character, these interactions can become the driving force in their physiological activity.

**Figure 3:**
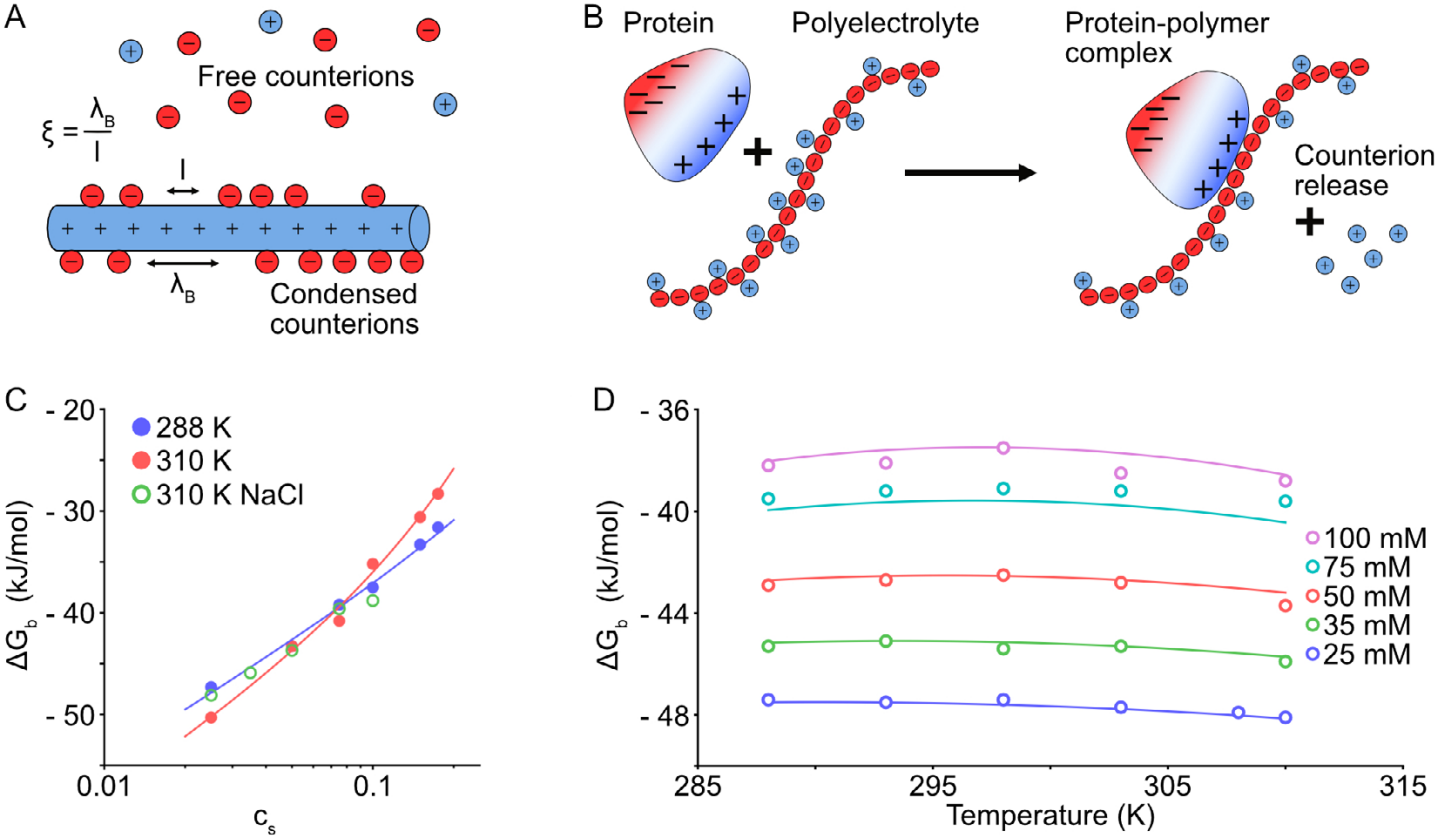
Charge–charge interactions and complex formation of proteins with polyelectrolytes. A) Characterization of linear polyelectrolytes by the charge parameter *ξ* (Equation 1).^[^ ^85^ ^]^ B) Counterion release during the complexation of a polyelectrolyte with a protein. C) Influence of hydration on complex formation. Dependence of Δ*G*_b_ on salt concentration *c*_s_ measured for the interaction of Hep with lysozyme in the presence of potassium glutamate at 288 K, near the characteristic temperature *T*_0_ (blue filled circles and fit) and at 310 K, above *T*_0_ (red filled circles and fit). The open green circles display the dependence of Δ*G*_b_ on *c*_s_ in the presence of NaCl.^[^ ^91^ ^]^ D) Δ*G*_b_ of the Hep–lysozyme complex formation plotted against *T* in function of *c*_s_.^[^ ^87^ ^]^ The fitted model takes hydration into consideration.^[^ ^81^ ^]^

**Figure** **3** displays the charge‐based interactions of GAGs with proteins.^[^ ^81^, ^83^ ^–^ ^84^ ^]^ In a simple model, a polyelectrolyte chain interacts with counter‐ and co‐ions (Figure 3A). The charge density of a GAG is characterized by the charge parameter:

(1) $$\begin{array}{c} \xi =\frac{\lambda_{B}}{l} \end{array}$$

where *l* is the distance between charges along the polyelectrolyte chain and *λ*_B_ is the Bjerrum‐length. The Bjerrum‐length is defined as

(2) $$\begin{array}{c} \lambda_{B}=\frac{e^{2}}{4\pi \varepsilon_{0}\varepsilon kT} \end{array}$$

with *e* denoting the elementary charge, *ε*_0_ the vacuum permittivity, *ε* the dielectric constant, *k* the Boltzmann constant, and *T* the temperature.

Based on Equations (1‐2), the extent of counterion condensation can be defined, which is widely supported by experimental evidence. If *ξ* > 1, a 1—1/*ξ* fraction of counterions is condensed onto the polyelectrolyte chain (‘Manning condensation’).^[^ ^85^ ^]^ These condensed counterions do not contribute to the osmotic pressure to the same extent as unbound species: the measured osmotic coefficient, that is, the fraction of osmotically active counterions agrees with predictions based on the Manning theory.^[^ ^85^ ^,^ ^86^ ^]^

Table 1 summarizes the charge parameters for all GAG classes from various sources. Hep has the highest degree of sulfation, which also leads to the highest charge parameter among GAGs. At pH = 7.4, that is, under physiological conditions, all carboxyl groups are deprotonated,^[^ ^87^ ^]^ which maximizes the charge parameter of Hep. This also makes Hep one of the most negatively charged biopolymers on Earth.

Most proteins exhibit an unevenly distributed surface charge, and positive patches may appear even above the isoelectric point. Many proteins that interact with Hep carry such positive patches.^[^ ^88^ ^,^ ^89^ ^]^ These (often sequence‐unspecific) Hep binding sites are discussed in detail with the conclusion that the mere presence of positively charged amino acids on a protein surface already ensures polyelectrolyte binding.^[^ ^90^ ^]^ The main features of a protein‐polyelectrolyte binding event are shown in Figure 3B: the binding leads to the partial release of condensed counterions, and the resulting entropy gain is a major driving force for complex formation. Since the condensed counterions can be considered chemically bound, the complex formation can be formulated in terms of a chemical reaction between the protein *P* and the polyelectrolyte *PE*:

(3) $$\begin{array}{c} P+PE⇌PEP+\text{Δ}n_{\mathrm{ci}}M^{+} \end{array}$$

where *PEP* denotes the complex and Δ*n*_ci_ the number of the released counterions *M* ^+^ *.* Since the number of salt ions in the system is much larger than *P* and *PE*, *[M* ^+^ *]* can be considered equal to the salt concentration *c*_s_ in the system.

These clearly imply that complex formation eventually depends on two decisive factors: the salt concentration *c*_s_ and temperature *T*. This is shown in Figure 3C‐3D with the experimentally determined free energy Δ*G*_b_ of complex formation between Hep and lysozyme.^[^ ^81^ ^,^ ^91^ ^]^ If counterion release dominates the complex formation, a logarithmic dependence of Δ*G*_b_ on *c*_s_ arises (Figure 3C, open green circles). Changes in hydration^[^ ^81^ ^]^ related to the Hofmeister effects lead to a slightly non‐linear dependence (Figure 3C, filled red circles). In the Hep–lysozyme interaction in the presence of potassium glutamate,^[^ ^91^ ^]^ the Hofmeister‐effects are much stronger than in the presence of NaCl, and they scale linearly with salt concentration, becoming prevalent at higher *c*_s_ *.*

The importance of charge–charge interaction in protein binding is well represented by the fact that Hep interacts with the largest number of proteins among GAGs.^[^ ^57^ ^,^ ^92^ ^]^ In numerous cases, the interaction of Hep with proteins is dominated by electrostatic effects,^[^ ^93^, ^94^, ^95^, ^96^ ^–^ ^97^ ^]^ with further in‐depth research fully corroborating it.^[^ ^81^ ^,^ ^87^ ^,^ ^91^ ^,^ ^98^ ^,^ ^99^ ^]^

An important prediction of the counterion release model is related to finite polyelectrolyte chain length. The model predicts^[^ ^100^, ^101^ ^–^ ^102^ ^]^ that counterion condensation only takes place if the polyelectrolyte chain exceeds a minimum length, which is also supported by experimental results.^[^ ^35^ ^]^ In a notable example, the dissociation constant of a complex between a synthetic peptide with Hep increases by one order of magnitude with a tetrasaccharide as compared to full‐chain Hep.^[^ ^93^ ^]^ Often, however, there are discrepancies between the predicted and measured values that cannot be explained by solely experimental difference. A probable explanation is the nonuniform counterion distribution along the polyelectrolyte chain, which was described in detail for various cases. For GAGs, the main source of deviation is reduced counterion density at the chains’ termini.^[^ ^102^ ^]^ Quantitative analyses of these end‐group effects in counterion condensation helped justify the experimental data,^[^ ^98^ ^,^ ^99^ ^,^ ^103^ ^]^ and they showed that Hep oligomers interact with proteins in a strongly Hep‐size‐dependent manner.^[^ ^104^ ^]^ More precisely, the magnitude of the end‐group effects scales with the reciprocal chain length of Hep,^[^ ^93^ ^,^ ^98^ ^]^ which agrees with earlier predictions.^[^ ^105^ ^]^

Evidently, the charge parameter *ξ* Equation (1) is an essential property of GAG chains, and a reduced *ξ* can lead to much weaker interactions. Still, the counterion condensation model is so far often neglected in the discussion of GAG‐protein interactions. Despite its underrepresentation, the model has strong implications, and a direct correlation can be drawn between the degree of sulfation and the electrostatic interactions of GAGs with proteins.^[^ ^57^ ^,^ ^92^ ^,^ ^106^ ^,^ ^107^ ^]^ With the increasing consideration of the role of counterion release as an entropic driving force of GAG–protein interactions, the next logical step is to understand how it translates to cases where specific GAG sequences interact with their binding sites.

## 5 Modified GAGs

GAG functional group modifications enable diverse applications. GAGs contain hydroxyl, carboxyl, *N*‐acetyl‐ (or amino‐), and sulfate groups, along with an aldehyde at the reducing end. In principle, each group can be selectively modified or eliminated. The distribution of modifications depends on the targeted functional group. For example, the aldehyde is unique because, regardless their size, each GAG molecule has only one reducing end. In contrast, carboxyl groups are homogeneously distributed along the chain, allowing for more homogeneous functionalization, while sulfate group density varies with high‐ and low‐sulfated motifs alternating along the chain.^[^ ^108^ ^]^

Modified GAGs have two primary applications: bioengineering and improved structural analysis. In bioengineering, Hep desulfation and HS (over)sulfation are key modifications, as the sulfation pattern directly relates to both the electrostatic and structural aspects of Hep interactions. Specific Hep desulfation alters its interaction with proteins,^[^ ^109^ ^,^ ^110^ ^]^ for example, with growth factors.^[^ ^111^ ^,^ ^112^ ^]^ As Hep and HS modifications affect protein binding, they ultimately regulate physiological and pathological responses. This leads to practical applications, such as the study of specific protein interactions, where desulfation can help identify specifically binding GAG sequences,^[^ ^113^, ^114^ ^–^ ^115^ ^]^ thus highlighting the structure–function relationship of Hep molecules in detail; for example, 2‐*O*‐desulfated Hep shows reduced binding to Factor H, which relies on multiple sulfate groups, while its interaction with FHR1, relying mostly on *N*‐sulfation, remains unaffected.^[^ ^115^ ^]^ In studies with histone acetyltransferase, both native and partially desulfated Hep inhibit enzyme activity, which suggests that their interaction is based on binding modes less affected by the degree of sulfation alone.^[^ ^116^ ^]^

Different from naturally sulfated GAGs, the modification of HA with sulfate groups introduces entirely new functional capabilities. For instance, sulfated HA resists degradation by hyaluronidase,^[^ ^117^ ^]^ including the inhibition of hyaluronidase CEMIP‐mediated degradation.^[^ ^118^ ^]^ In addition, it has also been demonstrated that sulfated HA extends the retention time of growth factors through electrostatic interactions, thus it can contribute to the residence and bioavailability of protein‐based drug formulations.^[^ ^117^ ^,^ ^119^ ^]^

HA sulfation can also tune receptor binding and signaling. For example, sulfation at the C6 position blocks CD44 binding of sulfated HA, while it enhances P–selectin interaction through long‐range electrostatic effects.^[^ ^120^ ^]^ Meanwhile, the growth factor signaling of HA can be modulated by sulfation, with the resulting modified HA binding VEGF165 stronger than native CS and Hep molecules, leading to a stronger anti‐angiogenic effect.^[^ ^121^ ^]^

Functionalized GAGs serve as a bridge between synthetic and natural polymers. They offer biocompatibility while retaining chemical tunability, which other biopolymers often lack. GAGs can be modified to form hydrogels that serve as scaffolds and anti‐inflammatory layers in tissue engineering. Hydrogels formed by crosslinking thiolated^[^ ^122^ ^,^ ^123^ ^]^ or transesterified GAGs^[^ ^121^ ^]^ exhibit diverse properties depending on the degree of modification, conjugate concentration, and crosslinking to other GAGs or synthetic polymers.^[^ ^124^ ^]^ When more specific GAG properties are of interest, for example, sequence specificity in interactions, they may need to be immobilized in a well‐defined manner. This can be achieved by functionalizing the reducing end with a linker, which facilitates detailed studies of GAG interactions.^[^ ^125^ ^]^

Structural modifications can also improve GAG detection and analysis. After their digestion into disaccharide units, GAGs can be labeled at the reducing end with small molecules to facilitate detection. Since disaccharides lack retention in reverse‐phase HPLC, acetylation^[^ ^126^ ^]^ or fluorescent labels, for example, 2‐aminoacridone, can help monitor their elution.^[^ ^127^ ^]^ These labels can have a secondary effect as well, for example, in ion mobility spectroscopy, where procainamide labeling improves GAG disaccharide separation.^[^ ^74^ ^]^ Other modifications, like permethylation, also contribute to GAG analysis, because they can provide more diagnostic fragments in tandem MS and prevent sulfate loss in fragmentation.^[^ ^128^ ^]^ This is particularly beneficial, since in most cases, the precise sulfation pattern is vital for understanding specific GAG binding. Thus, permethylation maximizes the structural information obtained from MS.

## 6 GAG Mimetics

Even though native and modified GAGs show high potential in therapeutics and other biomedical fields, their application comes with certain drawbacks. GAGs are highly heterogeneous, and natural products exhibit batch‐to‐batch variations. This also means that it is more difficult to exclude contamination.^[^ ^129^ ^,^ ^130^ ^]^ Hep, commonly extracted from porcine mucosa, may exhibit contamination with other GAGs or viruses, which can lead to severe side effects upon its clinical administration.^[^ ^33^ ^,^ ^131^ ^,^ ^132^ ^]^ However, high‐purity Hep can also lead to immune reactions by causing heparin‐induced thrombocytopenia and thrombosis (HITT), which induces activation and clotting of platelets, leading to low platelet levels in patients.^[^ ^26^ ^,^ ^133^ ^,^ ^134^ ^]^ Therefore, alternative anticoagulants, for example, smaller analogs like the synthetic glycopentamer Fondaparinux are used to avoid HITT.^[^ ^135^ ^]^ This, however, leads to orders‐of‐magnitudes reduction in anticoagulant activity as compared to unfractionated Hep (UFH).

The total synthesis of GAGs is difficult and often unprofitable; for example, the synthesis of the active pentamer sequence of Hep initially required more than 60 synthesis steps with 1% or less yield.^[^ ^136^ ^]^ Nowadays, this can be promoted by the implementation of automated solid‐state synthesis, allowing for fewer synthesis steps and higher glycooligomer yield.^[^ ^137^ ^,^ ^138^ ^]^ The products are highly similar to their natural counterparts and their structure can be precisely controlled, which paved the way toward new fields of applications for GAGs, fore example, as catalysts in chemical reactions.^[^ ^4^ ^]^ In certain cases, however, such as when long, precisely controlled structures are needed, this synthetic approach may become too complex for the goal.

GAGs can also be synthesized biochemically by enzymatic synthesis.^[^ ^139^ ^,^ ^140^ ^]^ It was shown that HS with different sulfation domain lengths can be chemoenzymatically generated, and the resulting GAGs can be used for assessing binding affinities to their natural interaction partners, such as chemokines.^[^ ^26^ ^,^ ^140^ ^]^ Together with recombinant technologies,^[^ ^141^ ^]^ which help access a large variety of GAGs and proteoglycans including their modifications, an extensive library of GAGs with precise structural control becomes available. However, in numerous cases, the efforts of producing pure GAGs outweigh the advantages of using bioidentical structures; here, GAG mimetics that are easier to produce in high quantities with well‐defined sequence and shape may be favored.

**Figure 4:**
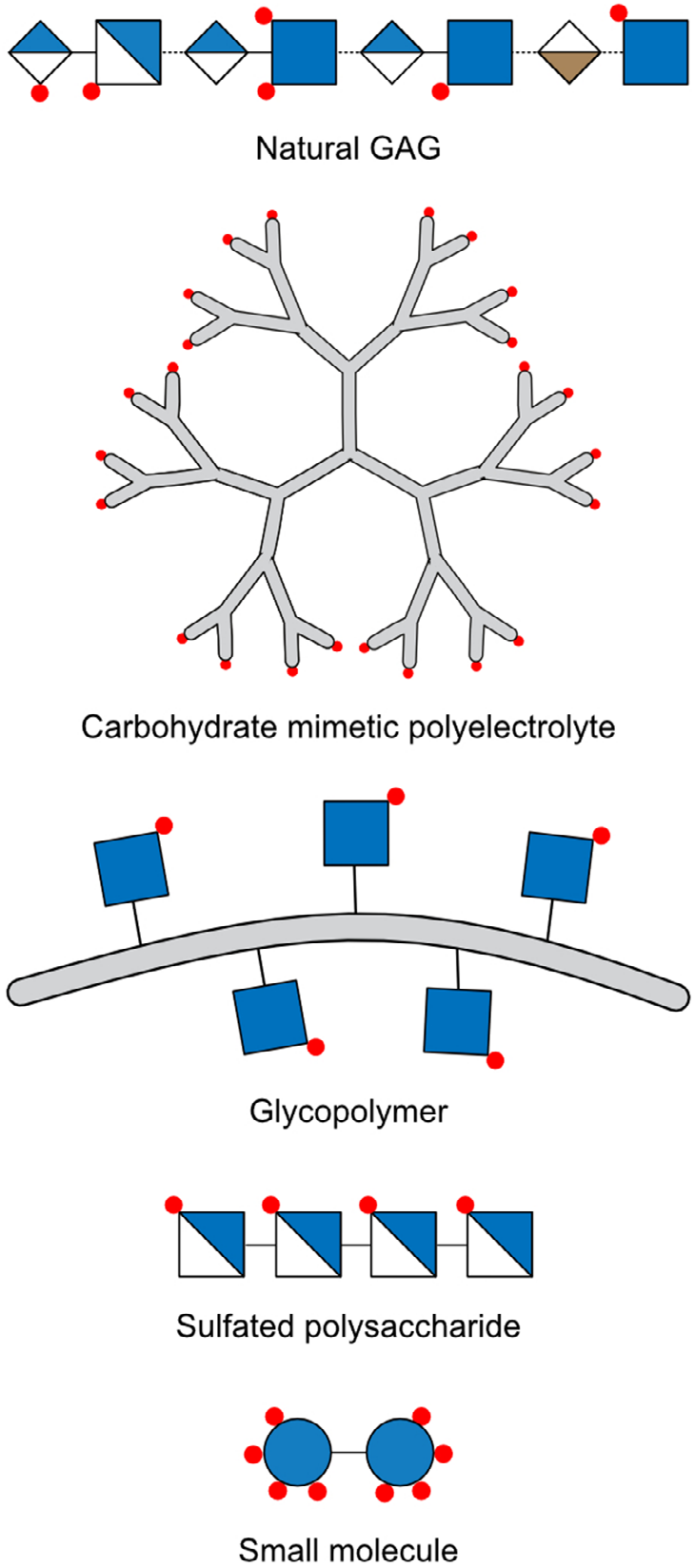
Different categories of a GAG molecule and synthetic mimetics. From top to bottom, a natural GAG structure (Hep) is compared to a carbohydrate mimetic polyelectrolyte (dendritic polyglycerol sulfate^[^ ^32^ ^]^), a glycopolymer^[^ ^146^ ^]^ with 6*‐* *O*‐sulfated GlcNAc, a sulfated polysaccharide (chitosan sulfate^[^ ^201^ ^]^), and a small molecule (octasulfated trehalose^[^ ^159^ ^]^).

As **Figure** **4** demonstrates, GAG mimetics can take different approaches with varying degrees of structural complexity and similarity to their natural counterpart. A simple and straightforward route is to modify other polysaccharides with sulfate‐ and carboxyl groups.^[^ ^142^ ^]^ Exopolysaccharide‐producing bacteria provide a biosynthetic alternative by secreting unsulfated polysaccharides that are structurally similar or even identical to GAGs.^[^ ^143^ ^]^ The isolated molecules can be chemically sulfated; such products show GAG‐like properties and can induce tissue regeneration or inhibit malignant cell migration.^[^ ^144^ ^,^ ^145^ ^]^

In more synthetic approaches, it is possible to synthesize glycopolymers with, for example, a methacrylate backbone and pendant sulfated and/or carboxylated carbohydrate units.^[^ ^146^ ^]^ The electrostatic interactions of these polymers can be tuned by copolymerizing monomers with sulfated and non‐sulfated pendant carbohydrates or other, biocompatible side groups, such as oligo(ethylene glycol) or amides.^[^ ^147^ ^,^ ^148^ ^]^ Due to their synthetic nature, the architecture of these polymers can be controlled. The length and character of side chains influence the glycopolymer properties, which translates to their physiological activity.^[^ ^149^ ^,^ ^150^ ^]^ Furthermore, such bottlebrush architectures can mimic proteoglycans,^[^ ^151^ ^]^ which can help understand the role of GAG chains in membrane physiological events. These nonlinear GAG mimetics can often show significantly stronger, multivalent binding to the biological target, which can be exploited in specific applications.^[^ ^152^ ^]^ Polymers with alternative architectures, for example, sulfated bottlebrush or dendritic polymers, can influence the access to the charged moieties in the molecule, which directly determines their complexation behavior.^[^ ^153^ ^]^

However, GAG mimetics do not always rely on carbohydrates. Carbohydrate‐free polymeric structures map the charge density and charge distribution of the GAG of interest, which is often enough to exert the necessary effect.^[^ ^154^ ^,^ ^155^ ^]^ This can be particularly useful in antimicrobial applications, where large amounts of the polyelectrolytes need to be produced with strong control over the structure. It was shown that synthetic linear polyglycerol sulfates can electrostatically associate with SARS‐CoV‐2 proteins (mimicking host‐pathogen interactions *via* cell‐surface HS) and block the host cell entry of the virus more efficiently than Hep.^[^ ^32^ ^]^ In the study, the structural similarity proved to be essential, since the linear structures outperformed dendritic polyglycerol sulfates with similar molecular weight. Meanwhile, dendritic carboxylated polyglycerols assembled into sheet‐like supramolecular structures demonstrated superior SARS‐CoV‐2 inhibition both in vitro and in vivo, which highlights that exploring structures with higher structural and chemical deviation from the natural target can yield much better outcomes.^[^ ^156^ ^]^ These results further exemplify the gap between bioidentical versus biocompatible, biosimilar structures in pharmaceutical attempts, since improved therapeutic efficacy outweighs the interest in complete structural overlap with endogenous molecules. Other polymers, such as poly(styrene sulfate) and poly((meth)acrylate) homo‐ and copolymers have been investigated for mimicking the electrostatic behavior of GAGs.^[^ ^146^ ^,^ ^147^ ^,^ ^157^ ^,^ ^158^ ^]^ In some cases, small molecules composed of sulfated saccharides^[^ ^152^ ^,^ ^159^ ^]^ or negatively charged, non‐carbohydrate molecules^[^ ^160^ ^]^ are used as GAG mimetics. These molecules can interact with binding sites on various proteins (e.g., factors in the coagulation cascade)^[^ ^161^ ^]^ or assemble into larger, three‐dimensional structures that mimic GAG‐based scaffolds.^[^ ^162^ ^]^ Fiber‐like assemblies are especially interesting for the generation of scaffolds for 3D cell cultures or tissue regeneration.^[^ ^151^ ^,^ ^162^ ^,^ ^163^ ^]^

In summary, different types of GAG mimetics can be synthesized and applied for various purposes; the necessary degree of similarity to their natural counterparts depends on the specific application and the need for specific interactions. In many cases, mimicking charge density and the distance between charged units and regions (e.g., regions with high and low sulfation) are sufficient to induce a certain effect (e.g., multivalent binding). While molecular weight and degree of sulfation of the mimetics directly influence their activity and mechanism of action,^[^ ^164^ ^]^ the generation of complex three‐dimensional structures (branched polymers, dendrimers, and large‐scale assemblies) can further enhance affinity of the mimetics to their targets and mimic not only a GAG sequence, but complete proteoglycans.^[^ ^165^ ^]^

## 7 GAG Sequestration

Therapeutics and medical devices exploiting charge‐based sequestration are often inspired by the electrostatic interactions of GAGs. The anticoagulant activity of Hep relies on charge–charge interactions between a highly anionic pentasaccharide sequence and cationic binding sites on antithrombin (AT), a protein responsible for the inhibition of coagulation factors IIa (thrombin) and Xa.^[^ ^166^ ^,^ ^167^ ^]^ Besides UFH, clinically used Hep includes low‐ molecular weight heparin (LMWH), and fondaparinux, a synthetic analog that mimics the pentasaccharide AT‐binding motif. UFH is commonly used for intraoperative coagulation management and the treatment of acute thrombotic blockage (e.g., heart attack, stroke, embolism, etc.), while LMWH and fondaparinux are typically used for post‐operative and other anticoagulant treatments.

Protamine sulfate, a highly cationic, arginine‐rich 4–5 kDa histone is used to rapidly reverse Hep activity through the formation of a stable Hep–protamine complex.^[^ ^11^ ^,^ ^168^ ^,^ ^169^ ^]^ However, protamine performs with reduced efficacy with LMWH and it is completely unable to inhibit fondaparinux. Moreover, its toxicity and limited therapeutic dosing window before triggering paradoxical bleeding at higher dose encourage the search for alternative Hep reversal agents.^[^ ^170^ ^]^

To find suitable alternatives, the cause behind the negative side effects of protamine must be understood. A mechanism has been proposed that depends on the sulfation and molecular weight of Hep or Hep derivatives, which can underly the differences between the complexation of UFH and LMWH with protamine. UFH is highly sulfated, with high negative charge density, which is sufficient to form stable complexes with protamine based on electrostatic attraction forces. In comparison, LMWH exhibits higher diversity in charge density among the molecules within the same sample, as well as an overall reduced average charge density. This lower average charge density can explain reduced complexation with protamine, leading to subpar performance in LMWH neutralization.^[^ ^171^ ^]^ Meanwhile, the chain length directly relates to the flexibility of Hep molecules, which can be crucial in adapting a stable conformation during complexation.^[^ ^172^ ^]^ The persistence length of Hep, that is, the length at which a polymer is considered rigid, is 2.11 nm, corresponding to approximately four monosaccharide units.^[^ ^173^ ^]^ For UFH, this length equals to only a small portion of its full chain length, while in the case of LMWH, it converges to the average chain length in the sample. This translates to UFH being a much more flexible, thus adaptable molecule in complex formation, while LMWH is a rather stiff interaction partner. This conformational stiffness is proposed to impair the ability of LMWH to form the necessary number of ion pairs in its complex with protamine.^[^ ^172^ ^]^ Due to the fact that the chain length, charge density, and conformational flexibility are often dependent on one another, it is challenging to determine the most important feature for strong complex formation in natural products.

Several universal Hep reversal agents have been identified, and through detailed mechanistic analysis, their performance can likely be improved in the future.^[^ ^82^ ^,^ ^174^ ^]^ These molecules are inspired by the electrostatic interactions within Hep‐protamine complexes, and they are designed for more stable Hep sequestration with high biocompatibility. Cationic polypeptides, although a straightforward choice, show limited success in these attempts due to challenges in their biological sourcing and potential for immunogenicity.^[^ ^175^ ^–^ ^177^ ^]^ In contrast, synthetic agents often successfully reverse all types of Hep. A notable recent example is MPI 2, which was developed with a charge switching concept, promoted by a polycationic binding group that displays low charge density near physiological pH when unbound, but adopts highly charged states when bound to a polyanionic species.^[^ ^178^ ^,^ ^179^ ^]^ This example also sheds light on the thermodynamic driving forces of efficient sequestration: when the pH‐responsive polycationic binding group switches to a high‐charge state upon heparin binding, 8–10 counterions are released, effectively leading to Δ*S* > 0. In contrast, the static high charge of protamine (pI > 12) causes nonspecific platelet activation. The charge switching behavior of MPI 2 confers the observed high hemocompatibility relative to other synthetic polycationic agents, due to the lower charge state of unbound molecules. Thus, it shows minimal platelet activation and a wide therapeutic window in contrast to protamine.^[^ ^178^ ^]^ Similar charge‐based interactions are often exploited, for example, in the case of an aminoethoxy‐phenyl‐pyridinium‐modified g‐cyclodextrin (PyA‐γ‐CD) that inhibits UFH and LWMH.^[^ ^180^ ^]^ This molecule displays high zeta potential, good Hep activity reversal, low hemolysis, and favorable cell viability, which outperform protamine within a similar therapeutic dosing window.^[^ ^180^ ^]^

Universal Hep reversal agents exhibit tunable features, which enables target specificity while reducing nonspecific interactions with other biomolecules. Additionally, the PEGylation of drug scaffolds can strongly increase Hep binding specificity due to the combined effect of the steric repulsion of non‐targeted molecules and Donnan shielding.^[^ ^172^ ^]^ The selective binding of Hep can be further improved by carefully tuning the cationic binding groups based on their amine pKa, linker chemistry, chain length, PEG chain grafting density, and charge spacing. By selecting generally weakly basic binding groups and engineering low charge densities at physiological pH, the off‐target activity can be minimized.^[^ ^179^ ^]^

Porous polymer scavengers can also sequester Hep.^[^ ^181^ ^]^ These can be based on supramolecular organic frameworks (SOPs) and porous organic polymers (POPs), and they reverse Hep activity through an inclusion‐sequestration strategy. These porous polymer scavengers can electrostatically bind Hep, while their rigid backbone stabilizes the complex by preventing deformation. Similar to this, other approaches also benefit from the special structural traits of the Hep binding partner by creating, for example, caltrop‐like architectures, whose Hep‐reversal activity was already demonstrated in preclinical studies.^[^ ^182^ ^]^

**Figure 5:**
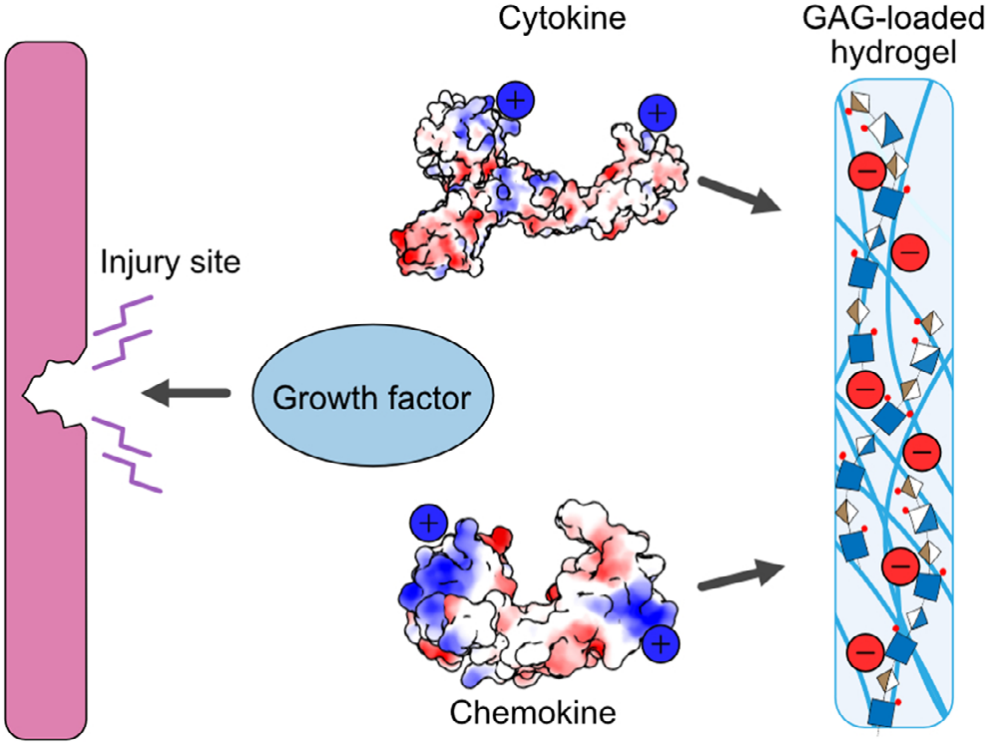
Schematic representation of the working mechanism of GAG‐based hydrogels in wound healing. While the assumed charges sequester cytokines and chemokines with strong positive patches, moderately charged species, for example, growth factors can remain at the site of injury.

The polyelectrolyte properties of GAGs are explored in other therapeutic areas as well, such as wound healing. During injury, a coordinated cascade of cytokines and growth factors are released to direct immune infiltration, inflammation, and subsequent healing.^[^ ^183^ ^]^ The functional principle of GAG‐like materials in these applications relies on their potential to influence cytokine gradients seen in physiological wound healing and inflammation.^[^ ^184^ ^]^ It is widely indicated^[^ ^23^ ^,^ ^184^, ^185^ ^–^ ^187^ ^]^ that anionic GAGs, specifically HS, are essential to establish these gradients by immobilizing cytokines near the site of injury, which results in their electrostatically mediated liquid–liquid phase separation on the cell surface.^[^ ^186^ ^]^ This gradient creates a haptotactic signal that drives immune cells to the site of injury. Hep@Gel, a photo‐crosslinkable gel of gelatine methacrylate (GelMA) and sulfhydryl‐modified Hep (HepSH) has been recently developed for scarless corneal wound repair. The gel is optically clear; the ratio of GelMA and HepSH help fine‐tune its mechanical properties,^[^ ^188^ ^]^ while the sulfate groups of Hep are responsible for scavenging IL‐1, TGF‐β, and PGDF‐BB. Presumably, the negatively charged HepSH component drives the immobilization of cytokines (including chemokines) by interacting with their basic amino acids, supported by studies on cytokine interactions with HS^[^ ^189^ ^]^ and Hep (**Figure** **5**).^[^ ^23^ ^,^ ^26^ ^]^ The electrostatic binding is confirmed, as the binding of matrix HepSH and cytokine is completely abated by immersion in 0.5 M KCl due to electrostatic shielding that is consistent with the Debye screening effect.^[^ ^190^ ^,^ ^191^ ^]^ Other chemokine scavengers, like multi‐armed poly(ethylene glycol) GAG (starPEG‐GAG) hydrogels were introduced in textile wound contact layers.^[^ ^192^ ^]^ By modulating charge densities in the hydrogel networks, CC, and CXC chemokines (e.g., IL‐8 and MCP‐1) can be sequestered, while the free movement of less‐charged growth factors associated with promoted healing remains.^[^ ^191^ ^]^

Additionally, polyelectrolyte hydrogels can be produced through electrostatically driven physical cross‐linking to form polyelectrolyte complexes (PECs) with a broad range of applications in wound healing.^[^ ^193^ ^]^ Chitosan/Hep sodium PEC gel films can be produced via self‐assembly, and they demonstrate pH‐dependent wound adhesion, favorable mechanical properties, and antibacterial activity.^[^ ^194^ ^]^ In a murine excisional wound healing model, these gels outperformed both negative and positive controls. With the additional possibility of complexing PEC gel components with antimicrobial metal ions, their antiseptic performance can be further enhanced.^[^ ^195^ ^]^ Low molecular weight polyanions enable the spontaneous mixing of PECs, and thus the dynamic modulation of the PEC size and shape *in situ*, which can have implications in enhanced targeting in wound healing applications.^[^ ^196^ ^]^

Recent discoveries demonstrate how fine‐tuning the electrostatic interactions between highly anionic GAGs and synthetic agents provides alternative biomedical approaches as well as a more detailed fundamental understanding of these interactions.^[^ ^176^ ^,^ ^178^ ^–^ ^181^ ^]^ These enable the rational design of molecules with high GAG specificity, exemplified by the above case studies on Hep neutralization and wound healing. Meanwhile, a more detailed understanding and optimization of these interactions, especially in GAG‐polycation complexes, could enhance their reliability and provide more effective translation into practical applications in the future.

## 8 Outlooks

Observing GAGs from a polyelectrolyte perspective provides a deeper insight into their complex physiological roles. As GAG exploration progresses,^[^ ^70^ ^]^ it becomes clear that their interactions with a wide variety of physiological species from simple metal ions to large protein complexes are central to proper cell functions. Placing the focus on these has enabled the understanding of cell signaling, structural support, host–virus interactions, and other key areas. However, due to analytical and sample limitations, our knowledge on GAGs as compared to other biopolymers, such as proteins or nucleic acids, is majorly lagging behind.

Looking ahead, there is significant untapped potential in expanding the applications of GAG‐based technologies, particularly in medicine and biotechnology. Modified GAGs and synthetic GAG mimetics are emerging as antiviral agents, offering new tools in the fight against rapidly spreading pandemics,^[^ ^32^ ^]^ and may help immune suppression as well as immune modulation in transplantation and cancer treatment.^[^ ^150^ ^,^ ^197^ ^,^ ^198^ ^]^ Furthermore, the ability to synergize GAGs with other therapeutic agents^[^ ^199^ ^]^ could lead to more effective treatments for cancer, neurodegenerative diseases, and other conditions where GAGs are critical.

From an analytical standpoint, the ongoing development of techniques to study GAGs at a molecular level will help decipher their behavior. Emerging mass spectrometry‐based techniques, especially those coupled with ion mobility‐mass spectrometry and cryogenic gas‐phase IR spectroscopy, will help access in‐depth structural features of these molecules, while also being adapted in routine GAG analytical workflows. Other advanced methods, such as 2D NMR and atomic force microscopy, are likely to be key in identifying subtle structural variations and understanding how these variations impact function. The continued refinement of these technologies will aid both fundamental research and the design of new biomaterials.

One of the greatest challenges remaining in the field is the limited knowledge of how GAGs influence cellular processes in vivo and to what extent. Therefore, it will be crucial to develop more sophisticated models to predict GAG behavior in biological systems, from their clearance rates in the bloodstream to their interactions with the cell membrane and the extracellular matrix in different tissues. These insights could pave the way for GAGs as therapeutic agents, delivery vehicles in targeted drug therapies, or the design of molecules with unique binding to GAGs in new treatment approaches.^[^ ^78^ ^]^

## Conflict of Interest

The authors declare no conflict of interest.
